# Characteristics of aquatic rescues undertaken by bystanders in Australia

**DOI:** 10.1371/journal.pone.0212349

**Published:** 2019-02-14

**Authors:** Robert W. Brander, Nicola Warton, Richard C. Franklin, Wendy S. Shaw, Eveline J. T. Rijksen, Shane Daw

**Affiliations:** 1 School of Biological, Earth and Environmental Sciences, UNSW Sydney, Sydney, New South Wales, Australia; 2 James Cook University, Townsville, Queensland, Australia; 3 Royal Life Saving Society Australia, Ultimo, New South Wales, Australia; 4 Surf Life Saving Australia, Bondi Beach, New South Wales, Australia; Seattle Children's Hospital, UNITED STATES

## Abstract

An issue of growing importance within the field of drowning prevention is the undertaking of aquatic rescues by bystanders, who sometimes drown in the process. The main objectives of this study were to describe characteristics of bystanders making rescues in different Australian aquatic environments, identify the role of prior water safety training in conducting bystander rescues and provide insights into future public education strategies relating to bystander rescue scenarios. An online survey was disseminated via various social media platforms in 2017 and gathered a total of 243 complete responses. The majority of bystander rescues described took place in coastal waterways (76.5%; n = 186), particularly beaches (n = 67), followed by pools (17.3%; n = 42) and inland waterways (6.2%; n = 15). The majority of respondents were males (64.2%; n = 156) who rescued on average approximately twice as many people in their lifetime (6.5) than female respondents (3.6). Most rescues occurred more than 1 km from lifeguard/lifesaver services (67%; n = 163), but in the presence of others (94.2%; n = 229). The majority of bystander rescuers had water safety training (65.8%; n = 160), self-rated as strong swimmers (68.3%; n = 166), conducted the rescue without help from others (60%; n = 146), did not use a flotation device to assist (63%; n = 153), but were confident in their ability to make the rescue (76.5%; n = 186). However, most considered the situation to be very serious (58%; n = 141) and felt they had saved a life (70.1%; n = 172). With the exception of pools, most bystanders rescued strangers (76.1%; n = 185).While Australia clearly benefits from having a strong water safety culture, there is no clear consensus on the most appropriate actions bystanders should take when confronted with a potential aquatic rescue scenario. In particular, more research is needed to gather information regarding bystander rescues undertaken by those without prior water safety training.

## Introduction

Drowning prevention is a complex global public health challenge with location, activity, age, gender, swimming ability, knowledge, environmental factors and proximity to rescue services all being factors that influence drowning outcomes [1–4]. The latter factor is particularly relevant as it is well documented that the vast majority of drowning fatalities occur in the absence of trained lifeguards or significant distances away from rescue and emergency services [2,5–8]. In these situations, the only form of help is often the presence of others who may act as bystander rescuers. The term ‘bystander’ describes any member of the public, be they family, friend or stranger, attempting to rescue someone in distress [9–10].

Bystanders are frequently involved in rescues in water environments and consequently play a significant role in saving lives [11–13]. The initial response and action of a bystander is often what saves lives, not only by rescuing someone from the water itself, but in assistance provided after the rescue, such as the application of CPR [11, 14]. However, the majority of bystanders are not trained or experienced in water-based rescue or medical assistance and often place both themselves and the rescuee(s) at risk while performing a rescue [9,15]. Unfortunately, it is also not uncommon for the bystander ‘rescuer’ to drown [10,12,16–18]. Furthermore, while every drowning is associated with significant emotional, societal and economic costs [19–20], drownings involving bystander rescuers often tend to receive more media exposure due to the altruistic and emotive nature of the rescue situation [21–24].

In 2014, the World Health Organisation (WHO) published ten actions to reduce the rates of drowning in their Global Drowning Report [2]. The fact that to “*train bystanders in safe rescue and resuscitation*” was fourth on the list is indicative of the recognition of the importance of the bystander rescuer issue. Previous studies on bystander rescues have explored the educational efficacy of water rescue safety messages, such as the 4 Rs of Aquatic Rescue: Recognise, Respond, Rescue and Revive [18]. The 4 Rs were based on the Drowning Chain of Survival [25] and when included as part of a water safety program, improved bystanders’ knowledge of safe rescue techniques [18]. Additionally, [26] assessed the effectiveness of someone throwing a lifeline, finding that training to throw a lifeline made a significant difference in the bystanders’ accuracy and throw distance; as a result, they recommended it as a critical skill for any potential bystander.

According to [13], recreational surfers along Australian beaches make as many rescues as professional lifeguards and most surfers involved in rescues felt their actions had saved a life. The advantage of surfers as bystander rescuers is that they have flotation devices (surfboards) and for the most part are competent swimmers. In contrast, in a New Zealand-based study, [12] assessed peoples’ readiness and capacity to form a rescue and found that nearly half (47%) of the survey respondents indicated they would jump in and attempt to save someone despite almost two-thirds (62%) identifying themselves as weak swimmers. Both cases highlight the need for bystanders to be prepared to react to, or undertake a rescue situation in the safest way possible. However, relatively few attempts have been made to formally document the actual experiences of bystander rescues and there is a paucity of research that quantitatively addresses the circumstances, characteristics and factors involved in both successful and unsuccessful bystander rescues [18].

An over-arching challenge in the field of drowning prevention, including the issue of bystander drowning, is the difficulty in obtaining accurate and quantitative information regarding drowning incidents [2] and while this problem is particularly acute in low- and middle-income countries (LMICs), it also applies to MICs like Australia, which has a well-established water safety industry and aquatic recreational culture [27–28]. According to the Royal Life Saving Society Australia (RLSSA), a total of 291 drowning deaths occurred across all Australian waterways between July 2016 and June 2017, a 4% increase on the previous 10 year average [29]. Inland waterways (e.g. rivers, lakes, dams) were the locations with the highest number of drowning deaths in 2016/2017 (33%) followed by beaches (17%), ocean/harbour locations (16%) and swimming pools (15%). While little is formally documented about the drowning deaths of bystander rescuers in these aquatic environments, a study of the Australian National Coroner’s Information System found that between 2002 and 2007, 17 rescuers in 15 separate incidents drowned whilst attempting to rescue a drowning child [9]. In 93% of these incidents the child, who initially required rescuing, survived. Of the drowning incidents, most were in ocean and beach environments.

Beaches in Australia patrolled by lifeguards and/or volunteer surf lifesavers are designated with one or more pairs of red and yellow flags denoting a supervised and safer swimming location. However, only 4% of the approximately 11,000 beaches in Australia are patrolled [7] and in 2016/2017, 74% of the 116 coastal drowning deaths occurred more than 1 km from the nearest beach lifeguarding/lifesaving service, either on unpatrolled beaches, or outside of patrol areas or times [7]. Some of these drownings involved bystander rescuers [23], which is symptomatic of an ongoing problem. According to Surf Life Saving Australia (SLSA) statistics, between 2004–2017, there have been 53 coastal drowning deaths of bystander rescuers on Australian beaches, representing 4% of all coastal drownings over this period (SLSA 2018, personal communication). Surf Life Saving Australia’s (SLSA) National Coastal Safety Survey 2017 [30] also reported that 13% of Australians in the 16–69 year old age bracket have acted as a bystander rescuer whilst participating in coastal activities.

While increasing the presence of lifeguard, lifesaving and emergency services in Australian (and global) aquatic environments is the most effective intervention to reduce drowning deaths [5–6], it is logistically impossible to have trained lifeguards ‘everywhere’ and at all times. Furthermore, apart from data collected through coronial investigations of drowning deaths, little is known about the circumstances of bystander rescues, particularly when bystanders survive the rescue. Details such as who the bystanders are (including their age, gender, swimming ability and training), as well as where, when and how the rescues are happening, what the bystanders’ reflections are on their rescue experiences, and what they would do differently next time, are all unknown and undocumented. This lack of information hinders the development of educational messaging to ensure the safety of people involved in bystander rescues.

### Aims

The primary aim of this study was to provide an evidence-based understanding of the occurrence and effectiveness of bystander rescues and their role in reducing aquatic drowning deaths in Australia. It attempted to understand the ‘who, where, when and how’ characteristics of bystander rescues and their frequency. Additionally, attempts were made to identify bystanders’ water competencies, associated rescue knowledge, and the role of flotation devices in rescues. A secondary aim was to assess the role of prior water safety training in conducting bystander rescues and examine the extent to which the existing water safety industry and culture in Australia influences bystander rescues The findings of this study will help build an accurate knowledge base for water safety practitioners to draw from in the provision of appropriate training of future bystander rescuers. This can only help reduce the incidence of fatal drowning deaths, both in Australia and globally.

## Materials and methods

To understand more about the circumstances surrounding bystander rescues in Australian aquatic environments, a convenience sample of people who had undertaken a bystander rescue was explored using an online Questionnaire Survey Instrument (QSI). The QSI was developed to collect both quantitative and qualitative data from bystanders about rescues they had undertaken in coastal and inland waterways and pool environments.

The ‘Citizen Lifesavers Survey’ contained 35 questions consisting of multiple choice, Likert Scale, number inputs and open answer questions and took approximately 10 minutes to complete. The survey contained three sections (Table 1): Section A: ‘About You’; Section B: ‘General Bystander Rescues’; and Section C: ‘Most Recent Rescue’ (Table 1). Sections A and B collected information about respondents’ demographics, water safety experience and training and number of rescues they had been involved in as a bystander across different types of aquatic environments. Coastal waterways were defined as bodies of water influenced by marine wave and tidal action (e.g. beaches, harbours, inlets, estuaries), inland waterways were bodies of freshwater (e.g. rivers, lakes, dams) and swimming pools were both public and private. Section C consisted of questions about the details about the respondents’ most recent rescue—such as their swimming ability, incident location, time of day, relationship with the rescuee, motivations for rescuing, how the rescue was performed and the outcome, and their reflections on the rescue; what they learned, their confidence levels and rescue capacity (Table 1). A full copy of the survey is provided in S1 Appendix.

**Table 1:** Examples of questions asked in the ‘Citizen Lifesavers Survey’. Note that some questions are slightly modified from versions in the actual survey (S1 Appendix).

**Section A: About You**	**Section C: Most Recent Rescue**
• What is your gender/age? • Which of the following best describes your swimming ability? • What water safety experience do you have?	• What kind of environment did your most recent bystander rescue occur in? (Pool/ Coastal/ inland water body) • At the time of the rescue, how far do you think you could swim in a pool without stopping? • At the time of the rescue, what experience did you have working in water safety? • Were there lifeguards or lifesavers on duty patrolling the area? • What were you doing at the time of the rescue? • What was the approximate age of the person? • What was your relationship to the person at the time of the rescue? • Did you use any devices to help you during the rescue, such as a surfboard or life jacket? • Would you do anything differently next time? If yes, what would you do differently?
**Section B: General Bystander Rescues**	
• As a bystander, how many people do you estimate you have rescued in all water environments in your lifetime? Which of the following water environments have you performed a rescue in as a bystander (*pool/coastal water body/inland water body*) Approximately how many rescues have you performed in each of these pool/coastal/inland waterway environments (*different options*)?	

The survey questionnaire focused on respondents’ most recent rescue, rather than most dangerous or most memorable, in order to collect responses from a range of different rescue scenarios that varied in severity. Additionally, memories of the most recent rescue are less likely to have been blurred over time. At the end of the survey, respondents were given the option to provide contact details for a follow up interview in order to collect more detailed information about their bystander rescue experience for a future study.

The ‘Citizen Lifesavers Survey’ was created using a UNSW Sydney based version of the survey software KeySurvey. Respondents were required to be a minimum of 18 years of age and were directed to a written Participant Information Statement at the beginning of the survey, which provided information about the survey including consent. In the case of active lifeguards or lifesavers, respondents needed to confirm that they were not on duty or patrolling at the time of the rescues they described in the survey. The UNSW Sydney Human Research Ethics Advisory Panel approved the survey under project number HC17298.

The survey was made available online on May 31^st^ 2017 and remained open until July 18^th^ 2017. Surf Life Saving Australia (SLSA), local surf lifesaving clubs, Royal Life Saving Australia (RLSSA), AUST Swim, UNSW Sydney and James Cook University promoted the online link. Personal social media networks of those involved in the study were also used. Media releases from UNSW Sydney and James Cook University generated some attention to the project via a number of radio interviews in New South Wales and Queensland. In total, the survey received 255 completed responses from bystander rescuers. Survey responses from respondents under 18 years of age and international respondents (outside Australia) were removed, leaving 243 Australian based responses for analysis.

Survey data was coded in line with the answer options available in the survey questions, except in a few cases where options were grouped due to low response numbers. Open-answer questions and those with long lists of answer options (such as age related questions) were simplified, and then coded (S2 Appendix). An example of the coding process relates to bystanders’ water safety training. In Section A of the survey, respondents were asked if they were currently working/volunteering in water safety and these responses were coded into two categories, ‘yes’ and ‘no’. ‘Yes’ included anyone who worked as a professional pool or ocean lifeguard, volunteered as a surf lifesaver, worked as a swim or surf instructor, or had a current Bronze Medallion (S2 Appendix). The Bronze Medallion is the core award to be a surf lifesaver in Australia (SLSA, 2015). Surf lifesavers are trained volunteers that patrol Australian beaches on weekends and public holidays during the extended summer season, often in addition to paid lifeguards. In Section C of the survey, respondents were asked what training they had at the time of their most recent rescue, and these responses were also coded into two categories: ‘none’ and ‘Water safety trained’. The latter included anyone who worked as a professional pool or ocean lifeguard, volunteered as a surf lifesaver or worked as a swim or surf instructor (S2 Appendix).

Once coded, data was imported into the statistical analysis software program R Studio (Version 1.0.143). Results were compared using contingency tables and tested for independence using Pearson’s Chi-square test. Throughout the analyses, a statistical significance level of 0.05 was used, where significance greater than 0.01, ‘p<0.01’ is used to describe the significance level. Details about respondents’ most recent rescue were compared with the location of the rescue, respondents’ gender, and their water safety training at the time of the rescue.

## Results and discussion

### Respondent details

The majority of survey respondents were male (64.2%; n = 156) and approximately half (50.6%; n = 123) of all respondents worked or volunteered in a water safety related role at time of survey completion. Most (76.4%; n = 94) of those working in water safety were male (χ^2^ = 16.2; p<0.01). In comparison, the gender breakdown of respondents not currently working or volunteering in water safety was almost equal (Table 2).

**Table 2:** Details about the bystander survey respondents at the time of completing the survey in relation to gender, age, number of rescues performed/type of waterway and if they were working in water safety. The total survey sample size was n = 243. Values in bold represent maximums for relevant categories. Shaded areas were found to be statistically significant.

	Bystander gender		Working in water safety	
	Male	Female	No (n = 120)	Yes (n = 123)
**Gender of respondents**				
Male	**156 (64.2%)**		62 (51.7%)	**94 (76.4%)**
Female		87 (35.8%)	58 **(48.3%)**	29 (23.6%)
**Current age of respondents**				
18–29 years	19 (12.2%)	19 (22.4%)	16 (13.3%)	22 (17.9%)
30–44 years	36 (23.1%)	**33 (38.9%)**	41 (34.2%)	28 (22.8%)
45–59 years	**74 (47.4%)**	24 (28.2%)	**43 (35.8%)**	**55 (44.7%)**
60+ years	27 (17.3%)	9 (10.6%)	19 (15.8%)	17 (13.8%)
**Total number of bystander rescues performed in lifetime (*n = 1317*)**				
Total	**1 008 (76.5%)**	309 (23.5%)	336 (25.6%)	**981 (74.5%)**
Average per respondent	**6.5**	3.6	2.8	**8.0**
**Average number of rescues within each aquatic environment**				
Coastal	**5.9**	**3.3**	**2.7**	**7.0**
Inland	1.8	1.5	1.4	2.0
Pool	2.8	1.9	1.5	3.3

At the time of completing the survey, respondents ranged from 18 to 84 years of age with the majority (68.7%; n = 167) aged between 30–59 years (Table 2). There was a strong association found between respondent age and gender (χ^2^ = 15.0; p<0.01), with the largest group of males and females aged between 45–59 years (47.4%) and 30–44 years (38.9%) respectively. Most respondents (71%) were from New South Wales (NSW) followed by Queensland (QLD; 11.5%), which is not surprising given the nature of the survey dissemination and the fact that NSW is Australia’s most populated state. The Northern Territory (NT) was the only State/Territory not to receive any responses.

### Involvement in bystander rescues

Section B of the survey asked respondents about their general involvement in bystander rescues across their lifetime. Respondents were asked approximately how many people they had rescued as a bystander and were then required to provide a breakdown of these numbers within different aquatic environments. On average, male respondents had performed almost twice as many bystander rescues than females (6.5 to 3.6; Table 2). Similarly, within each aquatic environment environment, the average number of rescues undertaken by males was greater than those by females (Table 2). Of note, respondents currently working in water safety had made almost three times as many bystander rescues (74.5%; n = 981) as those with no water safety training (25.6%; n = 336; Table 2). Across each aquatic environment, respondents working in water safety also performed on average more than twice as many rescues in pools (3.3 to 1.5) and coastal waterways (7.0 to 2.7) compared to those respondents without water safety training (Table 2).

### Most recent rescue details

Section C asked respondents about their most recent bystander rescue. The majority of rescues reported took place in coastal waterways (76.5%; n = 186), followed by pools (17.3%; n = 42) and inland waterways (6.2%; n = 15; Table 3). Within these locations, beaches were the most common coastal waterway (90%; n = 167), private pools the most common pool type (41%; n = 17), and rivers/ creeks the most common inland waterway (67%; n = 10).

**Table 3:** Details about the bystander and their most recent rescue. Values and percentages under the ‘Water Safety Training Column’ are based on sample sizes shown in left hand column (with the exception of ‘Gender of Bystander’). Values in bold represent maximums for relevant categories. Shaded areas were found to be statistically significant. Asterisks represent the significance level of the relationship, where * = p ≤ 0.05; ** = p ≤ 0.01 and *** = p ≤ 0.001.

	Bystander Gender		Water Safety Training	
	Male (n = 156)	Female (n = 87)	None	Yes
**Aquatic Environment**				
Coastal (n = 186)	129 (82.7%)**	57 (65.5%)**	59 (31.7%)**	127 (68.3%)**
Pool (n = 42)	18 (11.5%)**	24 (27.6%)**	22 (52.4%)**	20 (47.6%)**
Inland (n = 15)	9 (5.8%)**	6 (6.9%)**	2 (13.3%)**	13 (86.7%)**
**Gender of Bystander**				
Male (n = 156)			43 (27.6%) **	**113 (72.4%)** **
Female (n = 87)			40 (46.0%) **	**47 (54.0%)** **
**Age of bystander at time of rescue**				
0–17 years (n = 26)	15 (9.6%)	11 (12.6%)	14 (53.8%)**	12 (46.2%)**
18–29 years (n = 46)	30 (19.2%)	16 (18.4%)	12 (26.1%)**	34 (73.9%)**
30–44 years (n = 86)	48 (30.8%)	**38** (**43.7**%)	39 (45.3%)**	47 (54.7%)**
45–59 years (n = 72)	**51** (**32.7%**)	21 (24.1%)	15 (20.8%)**	57 (79.2%)**
60+ years (n = 13)	12 (7.7%)	1 (1.1%)	3 (23.1%)**	10 (76.9%)**
**Bystander pool swimming ability at time of rescue**				
Weak (n = 27)	10 (6.4%)	17 (19.5%)	**25 (92.6%)**	2 (7.4%)
Average (n = 50)	35 (22.4%)	15 (17.2%)	**24 (48.0%)**	26 (52.0%)
Strong (n = 166)	**111** (**71.2%**)	**55 (63.2%)**	34 (20.5%)	**132 (79.5%)**
**Time since rescue occurred**				
<6 months (n = 39)	22 (14.1%)	17 (19.5%)	8 (20.5%)***	31 (79.5%)***
6–12 months (n = 38)	29 (18.6%)	9 (10.3%)	5 (13.2%)***	33 (86.8%)***
1–2 years (n = 35)	22 (14.1%)	13 (14.9%)	9 (25.7%)***	26 (74.3%)***
3–5 years (n = 41)	22 (14.1%)	19 (21.8%)	18 (43.9%)***	23 (56.1%)***
5–10 years (n = 32)	23 (14.7%)	9 (10.3%)	11 (34.4%)***	21 (65.6%)***
>10 years (n = 58)	**38 (24.4%)**	**20 (23.0%)**	32 (55.2%)***	26 (92.9%)***

Although 64.2% (n = 156) of the bystander rescuers were male, the gender of the bystander rescuer was strongly associated with the location of the rescue (χ^2^ = 10.6, p<0.01) with males completing more coastal and inland waterway rescues than females, while females performed more pool rescues (Table 3). Approximately two thirds of the respondents (65.8%; n = 160) had been trained in water safety (e.g. professional beach/pool lifeguard, volunteer surf lifesaver, swim/surf instructor) at the time of performing their most recent rescue (Table 3). The remaining 34.2% (n = 83) had no water safety training or experience at all (Table 3). There was a significant relationship found between bystanders’ gender and their water safety training (χ^2^ = 8.4, p<0.01), with a higher proportion of males trained in water safety. Bystanders’ water safety training at the time of the rescue was also related to rescue location (χ^2^ = 9.6, p = 0.01), with more water safety trained bystanders performing coastal and inland waterway rescues (68.3%, n = 127; 86.7%, n = 13 respectively), while more untrained bystanders performed pool rescues (52.4%, n = 22; Table 3). Of note, most women who carried out pool rescues did not have water safety training.

Respondents were asked how old they were at the time of their most recent rescue and most were aged between 30 to 59 years (65%; n = 158). Within this age bracket, there was a similar number of male rescuers between the age groups 30–44 and 45–59 years, but more females aged between 30–44 years tended to make rescues (43.7%; n = 38; Table 3). Within the 30–59 age bracket, almost twice as many were trained in water safety (65.6%; n = 104) compared to those with no training (29.1%; n = 46; Table 3). Of note, bystander rescuers without any water safety training tended to be < 17 years of age or between the ages 30–44 (Table 3).

In general, the majority of bystander rescuers (68.3%; n = 166) self-rated themselves as ‘*strong*’ pool swimmers (able to swim more than 500 m without stopping) with the vast majority of ‘*weak*’ (92.6%, n = 25) swimmers (not able to swim 100 m) and ‘*average*’ (48%, n = 24) swimmers (able to swim 100–500 m) having no water safety training at the time of rescue (Table 3). Conversely, the majority of those with water safety training rated themselves as strong swimmers (79.5%; n = 132; Table 3). In terms of aquatic environment, 51% (n = 21) of pool rescues were performed by ‘*weak*’ or ‘*average*’ swimmers compared to only 28% of coastal rescues, which were dominated by strong swimmers with water safety training (Table 3). The distances used to define weak/average/strong swimmers were based on requirements associated with Australia’s national ‘Swim and Survive’ program and Bronze Medallion, which is the minimum standard required to be a lifesaver in Australia.

Respondents were asked how long ago their most recent rescue occurred and responses varied from ‘*< 6months*’ to ‘*10+ years*’ with a relatively even spread across all time brackets. However, while approximately a third of respondents (31.7%; n = 77) described recent rescues made within the last year, a somewhat higher proportion (37%; n = 90) described rescues that occurred more than 5 years previous (Table 3). A strong correlation was found between bystanders’ water safety training and time since the rescue occurred (χ^2^ = 24.9, p<0.01). Most rescues that occurred more than ten years previous were associated with bystanders without any water safety training (55.2%; n = 32), whereas 80.4% (n = 90) of rescues within the last 2 years were performed by bystanders with water safety training (Table 3).

Almost all rescues (91%; n = 221) occurred during ‘*daylight*’ hours between 8am and 8pm with no significant differences associated with waterway location. The majority of rescues in pools (69%; n = 29) and inland waterways (86.7%; n = 13) occurred in the complete absence of lifeguards and lifesavers (Table 4). In the case of coastal waterways, 64.5% (n = 120) of rescues took place greater than > 1 km from patrolled locations, outside of patrol hours, or on unpatrolled beaches (Table 4). Of note, the highest proportion of rescues carried out by bystanders without any water safety training (55.4%; n = 46) occurred in completely unpatrolled environments (Table 4).

**Table 4:** Details surrounding the context of the bystanders’ most recent rescue. Values in bold represent maximums for relevant categories. Shaded areas were found to be statistically significant. Asterisks represent the significance level of the relationship, where * = p ≤ 0.05; ** = p ≤ 0.01 and *** = p ≤ 0.001.

	Aquatic Environment			Bystander Gender		Water Safety Training	
	Coastal (n = 186)	Pool (n = 42)	Inland (n = 15)	Male (n = 156)	Female (n = 87)	None (n = 83)	Yes (n = 160)
**Presence of Lifeguard(s)/ Lifesaver(s)**							
Yes	42*** (22.6%)	11*** (26.2%)	1*** (6.7%)	33* (21.2%)	21* (24.1%)	16** (19.3%)	38** (23.8%)
<1 km away	24*** (12.9%)	1*** (2.4%)	1*** (6.7%)	21* (13.5%)	5* (5.7%)	7** (8.4%)	19** (11.9%)
> 1 km away	21*** (11.3%)	1*** (2.4%)	0*** (0.0%)	15* (9.6%)	7* (8.0%)	5** (6.0%)	17** (10.6%)
Outside patrol hours	50*** (26.9%)	0*** (0.0%)	0*** (0.0%)	39* (25.0%)	11* (12.6%)	9** (10.8%)	41** (25.6%)
None	49*** (26.3%)	29*** (69%)	13*** (86.7%)	48* (30.8%)	43* (49.4%)	46** (55.4%)	45** (28.1%)
**Bystander activity at time of the rescue**							
Swimming	62*** (33.3%)	20*** (47.6%)	6*** (40.0%)	49** (31.4%)	39** (44.8%)	**31** **(37.3%)**	**57** **(35.6%)**
Non powered watercraft	57*** (30.6%)	1*** (2.4%)	1*** (6.7%)	47** (30.1%)	12** (13.8%)	22 (26.5%)	37 (23.1%)
Walking nearby	27*** (14.5%)	3*** (7.1%)	1*** (6.7%)	21 (13.5%)	10 (11.5%)	11 (13.3%)	20 (12.5%)
Sunbathing	22*** (11.8%)	11*** (26.2%)	2*** (13.3%)	17** (10.9%)	18** (20.7%)	14 (16.9%)	21 (13.1%)
Boating	3*** (1.6%)	0*** (0.0%)	4*** (26.7%)	7** (4.abs5%)	0** (0.0%)	1 (1.2%)	6 (3.8%)
Dining nearby	5*** (2.7%)	3*** (7.1%)	0*** (0.0%)	5** (3.2%)	3** (3.4%)	1 (1.2%)	7 (4.4%)
Other	10*** (5.4%)	4*** (9.5%)	1*** (6.7%)	10** (6.4%)	5** (5.7%)	3 (3.6%)	12 (7.5%)
**Age of rescuee**							
0–9 years (*n = 6*2)	32*** (17.2%)	29*** (69.0%)	1*** (6.7%)	21*** *(33*.*9%)*	41*** *(66*.*1%)*	34** *(54*.*8%)*	28** (45.*2%)*
10–19 years (*n = 62*)	53*** (28.5%)	4*** (9.5%)	5*** (33.3%)	48*** *(77*.*4%)*	14*** *(22*.*6%)*	15** *(24*.*2%)*	47** (75.*8%)*
20–29 years (*n = 62*)	57*** (30.6%)	2*** (4.8%)	3*** (20.0%)	50*** *(80*.*6%)*	12*** *(19*.*4%)*	19** *(30*.*6%*	43** (69.*4%)*
30–39 years (*n = 19*)	14*** (7.5%)	1*** (2.4%)	4*** (26.7%)	11*** *(57*.*9%)*	8*** *(42*.*1%)*	4** *(21*.*1%)*	15** (78.*9%)*
40 + years (*n = 38*)	30*** (16.1%)	6*** (14.3%)	2*** (13.3%)	26*** *(68*.*4%)*	12*** *(31*.*6%)*	11** *(28*.*9%)*	27** (71.*1%)*
**Relationship to rescuee**							
Unknown	159*** (85.5%)	19*** (45.2%)	7*** (46.7%)	129*** (82.7%)	56*** (64.4%)	52*** (62.7%)	133*** (83.1%)
Known	27*** (14.5%)	23*** (54.8%)	8*** (53.3%)	27*** (17.3%)	31*** (35.6%)	31*** (37.3%)	27*** (16.9%)
**Rescuee had a flotation device**							
No	**161 (86.6%)**	**39 (92.9%)**	**11 (73.3%)**	**137 (87.8%)**	**74 (85.1%)**	**73 (88.0%)**	**138 (86.3%)**
Yes	25 (13.4%)	3 (7.1%)	4 (26.7%)	19 (12.2%)	13 (14.9%)	10 (12.0%)	22 (13.7%)
**Bystander used a flotation device to perform the rescue**							
No	95*** (51.1%)	39*** (92.9%)	9*** (60.0%)	80** (51.3%)	63** (72.4%)	56 (67.5%)	87 (54.4%)
Yes	85*** (45.7%)	2*** (4.8%)	2*** (13.3%)	66** (42.3%)	23** (26.4%)	26 (31.3%)	63 (39.4%)
Other	6*** (3.2%)	1*** (2.4%)	4*** (26.7%)	10** (6.4%)	1** (1.1%)	1 (1.2%)	10 (6.3%)

Respondents were asked about what they were doing before their most recent rescue took place. ‘*Swimming*’ was the most common activity in pools (47.6%; n = 20) and inland waterways (40%; n = 6), whereas both swimming (33.3%; n = 62) and using ‘*non-powered watercraft*’ (30.6%; n = 57) such as surfboards, paddle boards and canoes, were most common in coastal waterways (Table 4). Bystanders’ activity was associated with gender (χ^2^ = 16.8, p = 0.01) with significantly more males using ‘*non-powered watercraft’* and a higher proportion of females *‘sunbathing/ watching the water*’ at the time of the rescue (Table 4). Trends were similar regardless of whether the rescuers had water safety training or not.

Generally, bystanders’ most recent rescues in all aquatic environments took place in the presence of others. Most respondents reported that ‘*only a few*’ people were around (55.1%; n = 134) followed by ‘*lots of people*’ (39%; n = 95), particularly in inland waterway locations. There were only a few occasions (n = 14; 6%) across all aquatic environments when only the bystander and the rescuee were present at the time of rescue. There were no relationships between the number of people present and the gender or water safety training of the bystander rescuer. Despite most rescues occurring with other people around, most bystanders (60.1%; n = 146) performed the rescues without any help from others. There were no significant differences in this regard based on gender or water safety training. Where bystanders did have help, the average number of people who assisted across all aquatic environments was 1.8.

**Fig 1:**
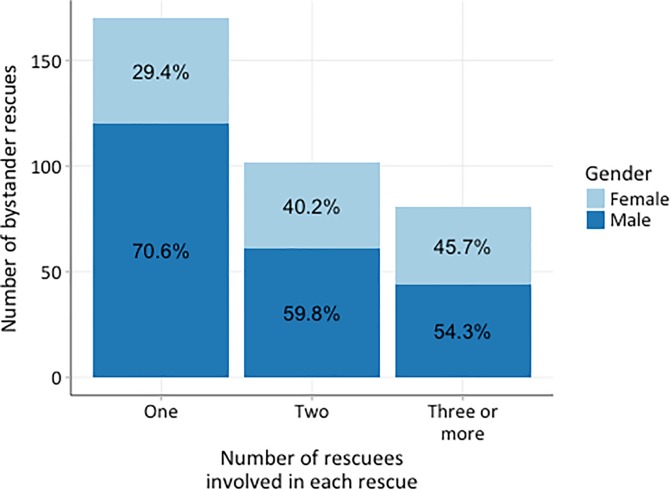
The number of rescuees involved in the bystanders’ most recent rescue. Gender differences are indicated based on the number of rescuees involved in the rescues.

A total of 353 people were rescued across all aquatic environments associated with the most recent rescues with almost twice as many males (63.7%; n = 225) being rescued than females (36.3%; n = 128; Fig 1). In general, 70% (n = 170) of the rescues involved only one rescuee, particularly in pools (88.1%; n = 37). The vast majority of rescues involving more than one person occurred in coastal waterways (86.3%; n = 63). There were four instances of mass rescues in coastal waterways involving more than 5 rescuees with the largest involving eight rescuees. There were no relationships between multiple rescues and gender or water safety training of the rescuer.

The majority of rescuees across all aquatic environments were aged between 0 and 29 years (76.5%; n = 186) with an equal number (n = 62; 25.5%) aged 0–9 years, 10–19 years and 20–29 years (Table 4). However, rescuees’ age was strongly related to aquatic environment (χ^2^ = 61.3, p<0.01), bystander gender (χ^2^ = 37.4, p<0.01), water safety training (χ^2^ = 31.7, p = 0.01) and relationship to the bystander rescuer (χ^2^ = 32.5, p<0.01). For example, the 0–9 year age group accounted for the majority (69%; n = 29) of rescuees in pools, more females (66.1%; n = 41) rescued this age group than males (33.9%; n = 21), and more untrained bystanders (54.8%; n = 34) rescued this age group than trained (45.2%; n = 28; Table 4). Of note, over half (53%; n = 31) of the ‘*known rescuees*’ were from within this age group.

Furthermore, the bystander’s relationship to the rescuee was also related to aquatic environment (χ^2^ = 38.2, p<0.01), bystander gender (χ^2^ = 10.3, p<0.01) and water safety training (χ^2^ = 12.6, p<0.01). While the majority of rescuees were mostly unknown to rescuers in coastal waterways (85.5%, n = 159), approximately half of bystanders making rescues in pools (54.8%; n = 23) and inland waterways (53.3%; n = 8) knew the rescuee (Table 4). Overall, a higher proportion of females (35.6%; n = 31) rescued people known to them than males (17.3%; n = 27; Table 4). Furthermore, bystanders who were untrained in water safety were more likely to rescue someone they knew (37.3%; n = 31) compared to bystanders with water safety training (16.9%; n = 27), who were more likely to rescue a stranger (83.1%; n = 133; Table 4).

Bystanders were asked how they came to be involved in their most recent rescue, with most (75.3%) saying it was when they ‘*saw someone in trouble and decided to help*’. In contrast, only 14% become involved because the ‘*person asked or signalled for help*’. These results were not associated with aquatic environment or the bystanders’ water safety training. Across all aquatic environments, most rescuees (86.7%; n = 211) did not have a flotation device with them when they needed rescuing (Table 4) although the proportion who did tended to be higher in inland waterways (26.7%; n = 4). There was no relationship between the rescuee having a flotation device and gender and water safety training of the bystander rescuer.

Across all aquatic environments, the majority of bystander rescuers did not use a flotation device to perform their most recent rescue (58.9%; n = 143), particularly in pools (92.9%; n = 39; Table 4). Use of a flotation device was associated with the aquatic environment of the rescue (χ^2^ = 45.6, p<0.01) and bystanders’ gender (χ^2^ = 11.5, p<0.01). For example, almost half of the bystander rescuers in coastal waterways (45.7%; n = 85) used a flotation device (Table 4). While the most common type of flotation device used in coastal waterways were surfboards/boogie boards, lifejacket and rescue tubes, respondents in inland waterways reported using a rope, tree branch or flippers (S3 Appendix). Across all aquatic environments, males were more likely (42.3%; n = 66) to use a flotation device than females (26.4%, n = 23; Table 4). Bystanders who had water safety training were also more likely to use a flotation device during the rescue (39.4%; n = 63) than those without training (31.3%, n = 26; Table 4), however this was not statistically significant. Most of the rescues (78.6%; n = 191) did not require any medical assistance for the rescuee. For those that did, 6.2% (n = 15) required First Aid, 1.6% (n = 4) required CPR and 13.6% (n = 33) involved an ambulance. Of note, most of First Aid (53.3%; n = 8) and ambulance cases (81.8%; n = 27) occurred in coastal waterways.

### Bystander reflections on their most recent rescue

The end of the survey included questions that asked respondents to reflect upon their most recent rescue experience. The first used a Likert scale that asked how serious the bystander thought the rescue situation was. Across all waterways the most common response was ‘*very serious*’ (58%; n = 141; Table 5). The seriousness of the situation was found to be strongly associated with the aquatic environment of the rescue (χ^2^ = 12.4, p<0.01) with 76.2% (n = 32) of pool rescues considered ‘*very serious*’ compared to 54.8% (n = 102) of coastal and 46.7% (n = 7) of inland rescues (Table 5). Similarly, seriousness of the situation was related to the condition of the rescuee after the rescue (χ^2^ = 28.7, p<0.01) as 90% of the rescues where the rescuee required medical assistance were rated as ‘*very serious*’. There was also a significant relationship between the seriousness of the rescue and water safety training (χ^2^ = 8.5, p = 0.02) with a higher proportion of untrained rescuers (65.1%; n = 54) tending to rate their rescues as ‘*very serious*’ compared to bystanders who had water safety training (54.4%, n = 87; Table 5).

**Table 5:** Bystanders’ reflections on their most recent rescue. Percentages are indicated in parentheses with those in italics related to sample sizes shown in italics. Values in bold represent maximums for relevant categories. Shaded areas were found to be statistically significant. Asterisks represent the significance level of the relationship, where * = p ≤ 0.05; ** = p ≤ 0.01 and *** = p ≤ 0.001.

	Aquatic Environment			Bystander Gender		Water Safety Training	
	Coastal (n = 186)	Pool (n = 42)	Inland (n = 15)	Male (n = 156)	Female (n = 87)	None (n = 83)	Yes (n = 160)
**Seriousness of the rescue**							
1–3: Not very	10* (5.4%)	1* (2.4%)	3* (20.0%)	8 (5.1%)	6 (6.9%)	8* (9.6%)	6* (3.8%)
4–7: Somewhat	74* (39.8%)	9* (21.4%)	5* (33.3%)	59 (37.8%)	29 (33.3%)	21* (25.3%)*	67* (41.9%)
8–10: Very	102* (54.8%)	32* (76.2%)	7* (46.7%)	**89 (57.1%)**	**52 (59.8%)**	54* (65.1%)	87* (54.4%)
**Confidence in their rescue ability**							
1–3: Not very	3 (1.6%)	1 (2.4%)	1 (6.7%)	1** (0.6%)	4** (4.6%)	4* (4.8%)	1* (0.6%)
4–7: Somewhat	36 (19.4%)	10 (23.8%)	6 (40.0%)	26** (16.7%)	26** (29.9%)	21* (25.3%)	31* (19.4%)
8–10: Very	**147 (79.0%)**	**31 (73.8%)**	**8 (53.3%)**	129** (82.7%)	57** (65.5%)	58* (69.9%)	128* (80.0%)
**Rescue difficulty**							
1–3: Not very	50** (26.9%)**	25** (59.5%) **	5** (33.3%) **	46 (29.5%)	34 (39.1%)	34 (41.0%)	46 (28.8%)
4–7: Somewhat	112** (60.2%) **	15** (35.7%) **	8** (53.3%) **	**93 (59.6%)**	**42 (48.3%)**	**39 (47.0%)**	**96 (60.0%)**
8–10: Very	24** (12.9%)	2** (4.8%)	2** (13.3%)	17 (10.9%)	11 (12.6%)	10 (12.0%)	18 (11.3%)
**Saved the rescuees’ life?**							
Yes	**130 (69.9%)**	**36 (85.7%)**	6 (40.0%)	**107 (68.6%)**	**65 (74.7%)**	**56 (67.5%)**	**116 (72.5%)**
No	47 (25.3%)	4 (9.5%)	**8 (53.3%)**	41 (26.3%)	18 (20.7%)	21 (25.3%)	38 (23.8%)
**Do anything differently next time?**							
No	**155 (83.3%)**	**36 (85.7%)**	**12 (80.0%)**	**133 (85.3%)**	**70 (80.5%)**	**66 (79.5%)**	**137 (85.6%)**
Yes	31 (16.7%)	6 (14.3%)	3 (20.0%)	23 (14.7%)	17 (19.5%)	17 (20.5%)	23 (14.4%)

Respondents were then asked to rate on a Likert scale their confidence in their ability to perform their most recent rescue. Across all aquatic environments most (76.5%; n = 186) said they were ‘*very confident*’ (Table 5), particularly in coastal and pool locations, but this relationship was dependent on gender (χ^2^ = 11.0, p<0.01) and water safety training (χ^2^ = 16.1, p<0.01). Proportionately more males (82.7%; n = 129) chose ‘*very confident*’ in their rescue abilities than females (65.5%; n = 57) and more bystanders with water safety training (80%; n = 128) chose ‘*very confident*’ than non-water trained (69.9%; n = 58; Table 5).

In terms of the difficulty in conducting their most recent rescue, only 11.5% (n = 28) of bystanders across all aquatic environments considered the rescue to be ‘*very difficult*’ (Table 5). However, rescue difficulty was related to aquatic environment location (χ^2^ = 16.8, p<0.01) as bystanders who performed coastal and inland waterway rescues rated their rescues as being more difficult (Table 5). For example, 56% (n = 136) of coastal waterway rescues and 66.7% (n = 10) of inland waterway rescues were rated as ‘*somewhat difficult*’ or ‘*very difficult*’ compared to only 40.5% (n = 17) of pool rescues (Table 5). There were no relationships between rescue difficulty and bystander gender and water training (Table 5).

Across all aquatic environments, the majority of bystander rescuers (70.8%; n = 172) thought their actions during their most recent rescue saved the life of the rescuee(s) (Table 5). This was strongly associated with the aquatic environment of the rescue (χ^2^ = 12.5, p<0.01), particularly in pools and coastal waterways where 85.7% (n = 36) and 69.9% (n = 130) of bystanders respectively felt they had saved a life (Table 5). There was a strong correlation between how the bystanders rated the seriousness of the rescue situation and if they felt they had saved the rescuees’ life (χ^2^ = 35.2, p<0.01). In general, 70% (n = 120) who said they thought they saved the life of the rescuee, rating the rescue situation as very serious.

**Fig 2:**
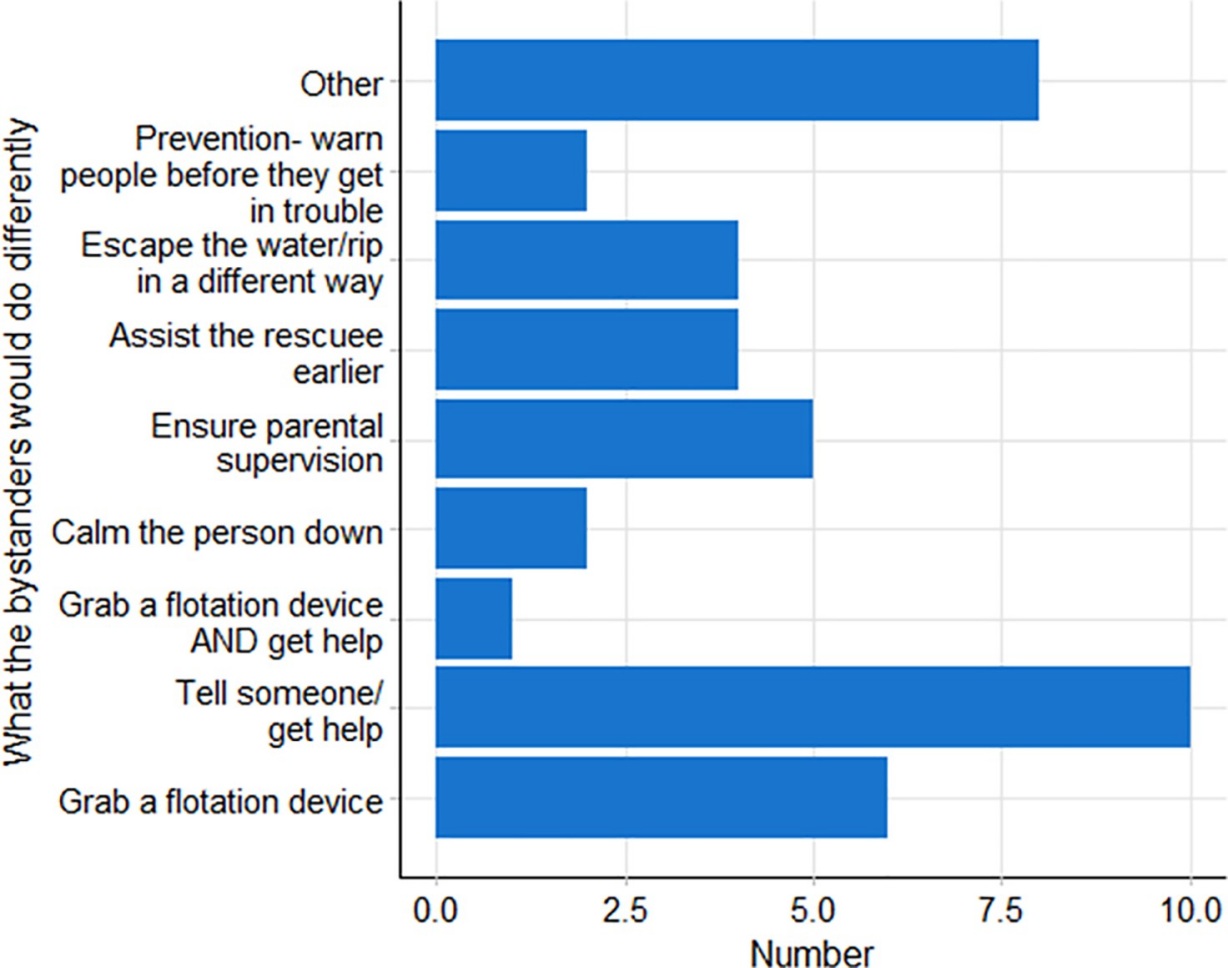
Actions that bystander rescuers’ indicated they would undertake differently if they had to perform a similar rescue in future (n = 40).

Bystanders were asked how they felt after their most recent rescue (S1 Appendix), with *‘happy to have helped*’ (68%; n = 166) and ‘*relieved*’ (47%; n = 114) being the most common responses. The final question in the survey asked bystanders if they would do anything differently next time, and if ‘*yes*’ what actions they would undertake. Regardless of aquatic environment, gender, and water safety training, most bystanders (83.5%; n = 203) said they would not do anything different (Table 5). The most common action for those who did (16.5%; n = 40) was that they would ‘*tell someone/get help*’ (24%, n = 10; Fig 2). However, it is clear from the variety of responses in Fig 2 that despite the small number of respondents to this question, there is no clear consensus regarding the most appropriate action to be taken in a bystander aquatic rescue scenario.

## Discussion

This study describes 243 cases of bystander rescues involving 353 people being rescued across coastal and inland waterways and pools in Australia. The significance of these rescues is evident from the fact that 71% (n = 172) of the bystander rescuers felt they had saved the life of the person(s) they rescued (Table 5). Assuming they were correct in this appraisal, this cohort of bystanders alone saved over 200 lives. Clearly, bystanders play a vital role in drowning prevention. However, while it is not possible to determine a total gross number or rate of occurrence of bystander rescues in Australian aquatic environments, the results presented in this study allow for profiles of bystander rescues to be described in regards to different aquatic environments as well as the gender and level of water safety training of the bystander rescuers. Here we highlight and discuss the key outcomes and implications of the study as well as some of the limitations and how future studies may be improved.

### General bystander rescue characteristics

The dataset was dominated by bystander rescues conducted in coastal waterways (76.5%; n = 186), of which 90% (n = 167) occurred on ocean beaches. This is perhaps not surprising given that an estimated 300 million beach visitations occur each year in Australia [31] and many beaches are characterized by dynamic wave, tide and current conditions [32]. However, this skewed sample size is also likely due to the nature of the survey dissemination, which was primarily through Surf Life Saving Australia (SLSA) and other coastal related social media platforms. Similarly, the proportion of bystander rescues in pools described in this study (17.3%; n = 42) was higher than those reported in inland waterways (6.2%; n = 15) due to the survey being promoted through AustSwim social media channels. AustSwim is Australia’s national organization for the teaching of swimming and water safety. It should therefore be noted that the dominance of bystander rescues in coastal waterways in this study is not representative of the overall drowning locations reported by [29], where 33% (n = 96) of the 291 drownings in Australia between July 2016 and June 2017 occurred in inland waterways.

Overall, most of the bystander rescuer respondents were male (64.2%; n = 156), which supports findings of other studies relating to bystander rescuers [11,16,33]. Most rescuers also had water safety training (65.8%; n = 160; Table 3) and while this finding is again likely influenced by the nature of the survey distribution via SLSA networks, it differs significantly from other studies of bystander rescues [10–11,16,18,33], which show the majority of bystander rescuers lack water safety training. Nevertheless, 34.2% (n = 83) of survey respondents in this study had no water safety training (Table 3), indicating that a large number of bystander rescuers in Australia are potentially placing themselves at risk due to a lack of formal water safety skills.

Most (67%; n = 163) rescues occurred more than 1 km from the nearest lifeguard/lifesaver patrol service or when and where none were present (Table 4). This value is similar to SLSA statistics for 2016–2017, which report that 65.5% (n = 76) of coastal drowning deaths occurred more than 1 km from the nearest lifesaving service [7]. While this emphasises the important role that bystanders play in the absence of lifeguards or lifesavers, it also highlights the risks involved in conducting rescues to the bystander with no proximal supporting emergency services in place to assist both during the rescue and in the case of any instances requiring medical assistance post-rescue. This risk is also enhanced for those bystanders without water safety training who made 36.8% (n = 60) of the rescues in these circumstances. Both risks are evident by the fact that SLSA statistics show that 74% (n = 53) of bystander rescuer drowning deaths in coastal waterways between 2004–2017 occurred in the absence of lifeguard/lifesaver services (SLSA 2018, personal communication).

The majority of bystander rescuers (75%; n = 182) acted on their own initiative suggesting that most bystanders self-identify when a rescuee requires assistance. This recognition of distress is a critical step in drowning survival, and was the second step in the *Universal Drowning Chain of Survival* by [25]. However, [34] showed that someone who was in significant distress and about to drown would be unlikely to be able to ask or signal for help, a factor that most bystanders not trained in water safety may not be aware of. An exception to this would be situations where the bystander rescuer knows the person in distress, particularly in the case of young children [9]. In this study, 24% (n = 58) of bystanders knew the person they rescued (Table 4). Of these, the highest proportion occurred in pools (54.8%; n = 23), most were female and most were untrained in water safety (Table 4). Unfortunately, it is not uncommon for parents and guardians to impulsively go to the aid of a drowning child, which can result in the drowning of the rescuer [9]. It has previously been suggested [9] that providing parents with personal life-saving skills is the most practical way to reduce such tragedies and the results of our study suggest that this type of training may be most useful if targeting female guardians.

Most of the bystanders performing rescues were between the ages of 30 and 59 (68.7%; n = 167; Table 3), which is consistent with bystander rescuer drowning data reported by SLSA between 2004–2017, where the highest percentage of bystander deaths (36%; n = 19) was in this age group (SLSA 2018, personnel communication). The majority of bystander rescues in this study also considered themselves to be strong swimmers (68.3%; n = 166; Table 3) and while the majority were very confident in their ability to perform the rescue (76.5%; n = 186), most found the rescue to be somewhat, or very, difficult (67.1%; n = 163) and considered the situation very serious (58%; n = 141), particularly those without any water safety training (Table 5).

The majority of bystanders (58.8%; n = 143) did not use flotation devices when conducting their rescue and the vast majority of rescuees (86.8%; n = 211) also did not have a flotation device (Table 4). However, bystanders with water safety training were more likely to use a flotation device during the rescue (70.8%; n = 63) than those who were not trained (29%; n = 26). This indicates the importance of having received water safety training in understanding that use of a flotation device can greatly assist in making a successful rescue, but it also highlights the need for communication of this particular message to the general (untrained in water safety) public. This is of particular importance given that none of the 53 bystander drowning deaths that occurred in Australia between 2004 and 2017 used a flotation device (SLSA 2018, personnel communication). This finding also supports those of [26], who identified the overwhelming assistance of lifelines, of some sort, in making successful rescues, and of [35] who found that the use of flotation devices saves time involved in rescue with positive repercussions in the reduction of drowning mortality and morbidity.

In terms of those rescued by the bystanders, the majority 63.7% (n = 225) were male. This value is consistent with the findings of the most recent Australian National Drowning Report [29] where 74% (n = 215) of all drowning fatalities between 2016 and 2017 were male. The over-representation of males, particularly young males, in rescue and drowning fatality statistics due to behavioral factors such as over-confidence and risk taking in aquatic environments is well documented [36–38]. However, while most of the rescuees in this study were between the ages of 0 and 29 (76.5%; n = 186; Table 4), the Australian National Drowning Report [29] reports that only 39.5% (n = 115) of drownings between 2016 and 2017 were over an equivalent age range. Most drowning deaths, particularly in coastal waters are traditionally over the age of 50 [7,29]. It is not clear why this trend does not appear in our data. In contrast, in the case of pool-based rescues, 69% the rescuees were children under 9 years old.

### Bystander rescues by waterway environment

The results of this study highlight several important differences between bystander rescues conducted in different aquatic environments (Table 6). Bystander rescues conducted in pools were noticeably different and were typically made by females without assistance from others (Table 4). This is largely explained by the fact that most of those rescued in pools were young children aged 0–9 years and were mostly known to the bystander rescuer, suggesting many of the rescuers were likely relatives or guardians of the rescuees. Most of the pool bystander rescuers also did not have water safety training, did not use a flotation device, and did not find the rescue very difficult, which is likely due to the proximity of the rescuer to the person in distress as well as calmer and shallower water conditions in pools.

**Table 6:** Characteristics and differences of bystander rescuers and rescues between Australian aquatic environments.

Bystander Rescuer/Rescue	*Pools*	*Coastal Waterway*	*Inland Waterways*
Gender	*Mostly female*	*Mostly male*	*Mostly male*
Water safety training	*Mostly no*	*Mostly yes*	*Mostly yes*
Lifeguard/Lifesaver presence	*Sometimes*	*Sometimes*	*Rare*
Age of rescuee	*Mostly < 9*	*Mostly 10–29*	*Mostly 10–19*
Relationship to rescuee	*Mostly known*	*Mostly strangers*	*Mostly known*
# Multiple rescuees?	*Rare*	*Relatively common*	*Relatively common*
Used a flotation device	*Mostly no*	*Even no/yes*	*Mostly no*
Rescue difficulty	*Not very*	*Somewhat*	*Somewhat*
Saved a life	*Strongly yes*	*Yes*	*Mostly no*

In contrast, most bystander rescues in coastal waterways were male, had water safety training, often used a flotation device (typically a surfboard/boogie board) and did not know the person they rescued (Table 6). Many coastal rescues involved assistance from other bystanders and were more likely to occur in the presence of lifeguards. The majority of rescues involving multiple rescuees also occurred in coastal waterways (Table 4). The age of those rescued ranged across all age groups, but were mostly between 10–29 years old. While it is difficult to draw strong conclusions regarding bystander rescues in inland waterways due to the small sample size (n = 15), it was rare for lifeguard services to be present and those rescued were mostly aged 10–19 years and were known to the rescuer (Table 6). Similar to coastal waterways, bystander rescuers often received help from others and often involved multiple rescuees.

### Implications of the study

Australia is somewhat unique in that it has a well-established volunteer surf lifesaving culture [28] initiated in the early 1900s and now has 311 surf lifesaving clubs consisting of 45,121 active patrolling members as of 2017 [7] all of whom are trained in water safety skills. This does not include the large number of trained professional lifeguard services on beaches around Australia. The results of this study clearly indicate the valuable role that off-duty surf lifesavers and lifeguards can play in drowning prevention as bystander rescuers, not only in coastal waterways, but in pools and inland waterways. Bystanders with water safety training had made three times as many rescues in their lifetime than those without any water safety training. Australia also has a large number of surfers who perform bystander rescues [13], a finding also apparent from results in this study. The importance of providing surfers with additional lifesaving skills is also recognized by the implementation of a program in Australia called ‘Surfers 24/7’ by Surfing Australia, which provides free lifesaving skills to surfers, including use of their board in the rescue and basic CPR skills.

However, as the results of this study show, many who perform bystander rescues in Australian aquatic environments do not have any water safety skills and for many the experience, even if successful, can be difficult both physically and emotionally. This is evident from the number of bystanders untrained in water safety who felt compelled to complete a survey recalling events that occurred more than 10 years ago. While it is impractical to assume that an entire population can be trained in water safety, the findings of this study suggest that various public messaging strategies relating to the issue of bystander rescues should be implemented. For example, many rescues took place in the presence of others, but the rescuers carried out the rescue by themselves. One message that could be promoted should be the importance of taking time to alert others to get help, which was the dominant action recommended by bystander respondents who suggested they would do something different if faced with the same situation again. This message is also relevant to weak swimmers, who were less likely to perform a rescue in coastal waterways, but presumably could alert others who were strong swimmers.

There is also evidence to suggest that different messages could be targeted to different aquatic environments. For pools the dominant message may relate to constant supervision of children and the importance of child guardians, particularly females, obtaining water safety skills. In the case of coastal and inland waterways, the results of this study suggest that the use of a flotation device is particularly important. However, many bystanders did not use a flotation device and did not, in reflection, consider this to be an action to be taken in the future. Promoting the importance to first look for a nearby flotation device (such as a boogie board) that could be provided to the person in distress, or assist the bystander in a rescue, may also help in achieving successful rescues and/or reducing the number of bystander rescuer drowning deaths.

However, the best form of intervention associated with any messages related to the issue of aquatic bystander rescues is prevention education at early ages, either during learn to swim classes, and/or at primary and high schools. The latter is particularly important as not all Australian school children receive swimming lessons, or are able to swim. In this case, messaging regarding bystander rescue scenarios that do not involve water entry is vital. This supports the drowning timeline of [39] where preparing communities at risk (i.e. potential bystander rescuers) is the first step in mitigating the drowning process. This preparation not only includes appropriate training about how to conduct a rescue, but educating about actions that should be taken before a potential incident occurs and learning how to react appropriately in a dangerous situation.

### Limitations and future improvements

This study involved an online survey and was therefore subject to responder bias and recall [40]. In particular, as described previously, the promotion of the survey through water safety social media channels and via localized media created a biased sample of respondents with water safety training and a limited geographic representation. As such, the results of this study are not representative of the general Australian population and cannot be generalized to other countries. Certainly, not many countries share the water safety culture (particularly the large number of trained lifesavers) that exists in Australia. Nevertheless, this study was a pilot for an intended larger study of aquatic bystander rescues in Australia and valuable lessons have been gained for improving future studies of this kind, particularly in terms of additional data that should be obtained.

A key data variable that should be asked/obtained from future bystander surveys is the cause of the incident leading to a rescue. As the focus of this study was on the bystander rescuers themselves, information on causal factors leading to the rescues was not captured. However, in the case of beaches, the main cause of lifeguard /lifesaver and surfer bystander rescues in Australia is due to people finding themselves caught in rip currents [13,41–43]. Rip currents are strong, narrow and fast flowing currents that exist on any beach characterized by waves breaking across a wide surf zone [44] and are ubiquitous features of many Australian beaches [45], particularly those in New South Wales where the majority of the survey respondents were from. There is no reason to believe that rip currents were not the main cause of the bystander rescues on beaches described in this study. Indeed, [13] found that 75% of bystander rescues performed by surfers were related to rip currents and SLSA statistics show that rip currents played a role in 70% of fatal bystander drowning incidents between 2004–2017 (SLSA 2018, personnel communication). Regardless, cause of rescue, particularly in inland waterways, is a critical data component for understanding the nature and risk involved in aquatic bystander rescues.

Other information that should be gathered in future studies relates to preventative actions that bystanders may have taken in the past to help others in aquatic environments before potentially dangerous incidents developed, or asking opinions on how they may conduct a preventative action in the future when given different scenarios. Elements of group behaviour, both in terms of the bystander rescuee and rescuer are also important in examining the behaviour leading to the incident and the reaction by the bystander rescuer. Finally, this study did not assess the exposure of the bystander rescuers and more information is needed on the frequency of visitation of bystanders to different aquatic environments and experience with difference forms of water-based recreational activities.

## Conclusions

The primary aim of this study was to provide an initial understanding of the ‘who, why, what, where, when, and how’ characteristics of Australian aquatic bystander rescues. In doing so it has shown the important role that bystanders, in particular those with water safety training, provide in saving lives. It has also provided insights into how the issue of bystander rescues and related bystander drowning deaths may be managed in the future, specifically through the need for public messaging to be developed and disseminated to the general Australian public. However, before this can be achieved further research is necessary to gather a larger dataset that encompasses all Australian regions, particularly inland waterways and coastal waterways other than surf beaches, as well as perceptions of those who have been rescued by bystanders.

## Supporting information

S1 Appendix Citizen lifesaver survey. (PDF)

S2 Appendix Recoding of survey data. (PDF)

S3 Appendix Actions that the bystanders indicated they would do differently next time they performed a rescue. (PDF)
