# Supplementary material for: Characteristics of aquatic rescues undertaken by bystanders in Australia
Source: PLoS One. 2019 Feb 14;14(2):e0212349. doi: 10.1371/journal.pone.0212349 (PMC6375621; doi:10.1371/journal.pone.0212349)
Supplement: S1 Appendix — (PDF) [file pone.0212349.s001.pdf]

Appendix 1: Citizen Lifesaver Survey

RED WORDING = Survey Logic

* = Compulsory Question

Introduction

- This survey is part of a study by UNSW Sydney, James Cook University and Surf Life Saving Australia looking at the involvement of bystanders in rescuing people from the water, such as in a river, at the beach, or in a pool, as well as the experiences of people rescued by bystanders.
- A *bystander rescuer* is someone, be that a member of the public, family or friend, who has seen or heard someone in the water, who needed help and rescuing, and has offered assistance. For the purpose of this study, an on duty lifeguard or lifesaver is NOT a bystander.
- A *bystander rescuee* is someone who has needed the help of a bystander to rescue them from the water.
- Bystander rescues range from a person jumping into a swimming pool to rescue a person, to a surfer, swimmer or other person helping a swimmer in difficulty.
- If you have ever worked or volunteered in water safety (e.g. as a lifeguard or a lifesaver) we want to hear about the incidents you have been involved in **outside of your paid or volunteer work (i.e. those outside of your patrolling hours)**.

Conditions of survey: *

I am over 18 years of age.

I recognise that this is a survey about bystander rescues. If I have ever worked or volunteered as a lifeguard or lifesaver I will answer the questions with regards to incidents that have occurred **while I have been off duty**.

○ I agree, start survey (***Q.1***)

○ I disagree (***Taken to end of survey***)

1. Have you ever rescued someone from water as a bystander? *
   a. Yes (***Taken to PART 1***)
   b. No (***Go to Qu. 2***)

2. Have you ever needed to be rescued from water? *
    a. Yes (***Taken to PART 2***)
    b. No (***Taken to end of survey***)

PART ONE: SURVEY FOR BYSTANDER RESCUERS

Section A: About You

The following questions are about you to help us understand what types of people undertake rescues.

1. What is your gender? *
    A. Male
    B. Female
    C. Other

2. How old are you? (years)(open)

3. What country do you currently live in?* (Drop down menu of countries) (***If Aus is selected, go to Q. 4***)

4. Which Australian state or territory do you currently live in? *
    A. New South Wales
    B. Queensland
    C. Victoria
    D. South Australia
    E. Western Australia
    F. Northern Territory
    G. Tasmania
    H. Australian Capital Territory

5. Are you currently working/ volunteering in water safety? (You can select more than one) *
    A. No
    B. Yes- Trained volunteer surf lifesaver
    C. Yes- Trained professional ocean lifeguard
    D. Yes- Trained professional pool lifeguard
    E. Yes- Trained swim instructor
    F. Yes- Trained surf instructor
    G. Other (please specify):

Section B: General Bystander Rescues

We now want to know about your experience with rescues across your lifetime.

6. As a bystander, how many people do you estimate you have rescued in all water environments in your lifetime? (Give approximate number) * (Open answer)

7. Which of the following water environments have you performed a rescue in as a bystander? (You can select more than one) *
    A. A pool

B. A <u>coastal</u> water body (e.g. a beach, the ocean or a marina)
C. An <u>inland</u> water body (e.g. a lake or a river)

Hover definitions:
Coastal= -'Areas affected by tidal movements and which have salt water.'
Inland= 'Areas that aren't affected by tidal movements.'

(***Whatever combination of options they pick here, the relevant table (Q.8-10) with those environments will appear next***)

8. Approximately how many rescues have you performed in each of these pool environments? (If none, write '0')

Public Pool (e.g. a council pool)	(*insert number >= 0*)
Private Pool (e.g. a backyard pool)	(*insert number >= 0*)
Hotel or Resort Pool	(*insert number >= 0*)
Other	(*insert number >= 0*)
**Total Number of Pool Rescues**:	*(Automatically sums total of above rescues)*

9. Approximately how many rescues have you performed in each of these coastal environments? (If none, write '0')

Beach (<500m from shore)	(*insert number >= 0*)
Rocky coast (e.g. shore platform, cliffs, rocky headland, reef)	(*insert number >= 0*)
Open ocean (500m or more from shore)	(*insert number >= 0*)
Jetty/ pier	(*insert number >= 0*)
Other	(*insert number >= 0*)
**Total Number of Coastal Rescues**:	*(Automatically sums total of above rescues)*

10. Approximately how many rescues have you performed in each of these inland water environments? (If none, write '0')

Lake	(*insert number >= 0*)
Dam	(*insert number >= 0*)
River or Creek	(*insert number >= 0*)
Other	(*insert number >= 0*)

Section C: Most Recent Rescue

These questions are asking you about your MOST RECENT rescue you have undertaken as a bystander (it may also be the only one you have ever done).

11. What kind of environment did your most recent bystander rescue occur in? *
    A. Pool (***Q.12***)
    B. Coastal water body (***Q.13***)
    C. Inland water body (***Q.14***)

12. What kind of pool environment? *

A. Public Pool (e.g. a council pool)
B. Private Pool (e.g. a backyard pool)
C. Hotel or Resort Pool
D. Other (please specify):

13. What kind of coastal water body? *
    A. Beach (<500 m from shore)
    B. Rocky coast (e.g. shore platform, cliffs, rocky headland, reef)
    C. Open ocean (> 500 m from shore)
    D. Jetty/ pier/ marina
    E. Other (please specify):

14. What kind of inland water body? *
    A. Lake
    B. Dam
    C. River or creek
    D. Other (please specify):

15. What country did the rescue take place? * (drop down menu) (***if Aus- go to 16, otherwise 17***)

16. Which Australian State or Territory did the rescue occur in? (drop down menu)
    i. New South Wales
    ii. Queensland
    iii. Victoria
    iv. South Australia
    v. Western Australia
    vi. Northern Territory
    vii. Tasmania
    viii. Australian Capital Territory

17. Approximately how old were you when you performed the rescue? (Open)

18. How long ago did the rescue occur?
    A. Within the last 6 months
    B. Between 6-12 months ago
    C. Between 1-2 years ago (13 to 24 months)
    D. Between 2-5 years ago (25 to 60 months)
    E. Between 5-10 years ago (61 to 120 months)
    F. More than 10 year ago

19. At the time of the rescue, how far do you think you could swim in a pool without stopping?
    A. I cannot swim
    B. Less than 25 m (about 25 yards)
    C. Between 25 and 100 m
    D. Between 100 and 500 m
    E. More than 500 m
    F. Unsure

20. At the time of the rescue, what experience did you have working in water safety? (You can select more than one) *
    A. None

B. Trained volunteer surf lifesaver
C. Trained professional ocean lifeguard
D. Trained professional pool lifeguard
E. Trained swim instructor
F. Trained surf instructor
G. Bronze Medallion
H. Other (please specify):

21. Were there lifeguards or lifesavers on duty patrolling the area? *
    A. Yes
    B. No, but there were lifeguards or lifesavers patrolling less than 1km away
    C. No, but there were lifeguards or lifesavers patrolling between 1 and 5km away
    D. No, but there were lifeguards or lifesavers patrolling more than 5km away
    E. No, it was outside of patrolled hours
    F. No
    G. Don't know

22. Approximately, what time of day did the rescue occur? *
    A. 12 am – 4 am
    B. 4 am – 8 am
    C. 8 am – 12 pm
    D. 12 pm -4 pm
    E. 4 pm – 8 pm
    F. 8 pm -12 am
    G. Unsure

23. What were you doing at the time of the rescue? *
    A. Swimming
    B. Surfing
    C. Walking/ running near by
    D. Watching the water
    E. Boating
    F. Canoeing, kayaking or paddle boarding
    G. Fishing
    H. Supervising kids swimming
    I. Dining nearby
    J. Other (please specify):

24. Were there people present when the rescue took place?
    A. Yes, it was busy with lots of people around
    B. Yes, but there were only a few people around
    C. Nobody was around except for me and the person in trouble
    D. I can't remember

25. Did other people help you with the rescue?
    A. No
    B. Yes
    If yes, how many? (Comment box)

26. How many people needed rescuing? * (Insert the number of each gender)
    A. Male (*insert number >= 0*)

B. Female (*insert number >= 0*)
C. Other (*insert number >= 0*)
Total number of people rescued: *(Automatically sums total of above rescues)*

Please tell us a little bit about the person you rescued. If you rescued multiple people, tell us about the first person you rescued at the time.

27. What was the approximate age of the person? * (Drop down menu)
    A. 0-4 years
    B. 5-9 years
    C. 10-14 years
    D. 15-19 years
    E. 20-24 years
    F. 25-29 years
    G. 30-34 years
    H. 35-39 years
    I. 40-44 years
    J. 45-49 years
    K. 50-54 years
    L. 55-59 years
    M. 60-64 years
    N. 65-69 years
    O. 70-74 years
    P. 75-79 years
    Q. 80-84 years
    R. 85+ years

28. What was your relationship to the person at the time of the rescue?
    i. Stranger
    ii. Parent
    iii. Brother/ Sister
    iv. Child
    v. Grandchild
    vi. Grandparent
    vii. Aunt/ Uncle
    viii. Cousin
    ix. Spouse/ Partner
    x. Friend
    xi. Other (please specify):

29. Did the person have any flotation devices with them at the time? *
    A. No
    B. I don't know
    C. Yes, they had (please specify):

30. How did you come to be involved in the rescue? *
    A. The person/s in trouble asked and/ or signaled for help
    B. I/ we saw the person/s in trouble and decided to help
    C. Someone else requested my/ our help
    D. Other (please specify):

31. On a scale of 1-10, how serious did you think the situation was? *

1. Not that serious, but the person/s needed a little bit of help
2.
3.
4.
5. Somewhat serious, the person/s needed help
6.
7.
8.
9.
10. Very serious, the person/s were going to drown

32. When you came to be involved in the rescue, on a scale of 1-10, how confident were you in your ability to rescue the person/s? *

1. Not at all confident
2.
3.
4.
5. Somewhat confident
6.
7.
8.
9.
10. Completely confident

33. Did you use any devices to help you during the rescue, such as a surfboard or life jacket? *

A. No
B. Yes- A surfboard
C. Yes- A life jacket
D. Yes- Other (please specify):

34. What was the person/s like when you got to them?

A. Calm
B. Relieved
C. Embarrassed
D. Panicked
E. Exhausted
F. Unconscious
G. I can't remember
H. Other (please specify):

35. How was the person/s after the rescue? * (Can select more than one)

A. Required CPR
B. Required First Aid
C. Required an ambulance
D. Ok, could walk away
E. I can't remember

36. Do you think you saved the person/s life?

a. Yes

b. No
Add a comment

37. On a scale of 1-10, how difficult was it for you to rescue the person/s? *
    1. Easy, it was not difficult at all
    2.
    3.
    4. Relatively easy but had a little bit of trouble
    5.
    6.
    7. Relatively difficult
    8.
    9.
    10. Very difficult, I really struggled

38. How did the experience leave you feeling immediately after the rescue? * (You can select more than one) *
    A. Happy to have helped
    B. Relieved
    C. Inconvenienced
    D. Annoyed
    E. Scared
    F. Sad
    G. Other (please specify):

39. Would you do anything differently next time? *
    A. No
    B. Yes
    If yes, what would you do differently? (Comment box)

Thank you for taking the time to fill out this survey about your rescue.

Have you also needed a bystander to rescue you from the water? *

- Yes and I'd like to fill out a survey about my experience (***Skips the 'About you' and goes to Q. 6 of Part 2)***
- No (***Contact details and end of survey***)
- I'm done (***Contact details and end of survey***)

Contact Details:

If you would like to be involved in further research about your bystander rescue in the future (such as an interview), please provide your contact details below:

Name:
Best contact detail (email/ phone number):

End of survey message: *Thank you for filling out this survey. Please share it with your friends.*
