# Supplementary material for: Characteristics of aquatic rescues undertaken by bystanders in Australia
Source: PLoS One. 2019 Feb 14;14(2):e0212349. doi: 10.1371/journal.pone.0212349 (PMC6375621; doi:10.1371/journal.pone.0212349)
Supplement: S2 Appendix — (PDF) [file pone.0212349.s002.pdf]

Appendix 2: Recoding of Survey Data

Survey Question	Code	Coded Category	Original Category
**How old are you? and How old were you at the time of the rescue?**	1	0-17 yrs	0-11 yrs, 12-17 yrs
	2	18-29 yrs	18-24 yrs, 25-29 yrs
	3	30-44 yrs	30-34 yrs, 35-39 yrs, 40-44 yrs
	4	45-59 yrs	45-49 yrs, 50-54 yrs, 55-59 yrs
	5	60+ yrs	60-64 yrs, 65-69 yrs, 70-74 yrs, 75-79 yrs, 80-84 yrs, 85+ yrs
**How old was the rescuee?**	1	0-9 yrs	0-4 yrs, 5-9 yrs
	2	10-19 yrs	10-14 yrs, 15-19 yrs
	3	20-29 yrs	20-24 yrs, 25-29 yrs
	4	30-39 yrs	30-34 yrs, 35-39 yrs
	5	40 + yrs	40-44 yrs, 45-49 yrs, 50-54 yrs, 55-59 yrs, 60-64 yrs, 65-69 yrs, 70-74 yrs, 75-79 yrs, 80-84 yrs, 85+ yrs
**Are you currently working/volunteering in water safety?** *[‘Other’ was recoded into the appropriate category]*	1	No	No
	2	Yes	Yes- Trained volunteer surf lifesaver, Yes-Trained professional ocean lifeguard, Yes-Trained professional pool lifeguard, Yes-Trained swim instructor, Yes- Trained surf instructor
**At the time of the rescue, what experience did you have working in water safety?** *[‘Other’ was recoded into the appropriate category]*	1	None	None
	2	Water Trained	Trained volunteer surf lifesaver, Trained professional ocean lifeguard, Trained professional pool lifeguard, Trained swim instructor, Trained surf instructor, Bronze Medallion
**At the time of the rescue, how far do you think you could swim in a pool without stopping?**	1	Weak Swimmer	Unsure, Can’t swim, Less than 25m, Between 25 and 100m
	2	Average Swimmer	Between 100 and 500m
	3	Strong Swimmer	More than 500m
**Were there lifeguards or lifesavers on duty patrolling the area?**	1	Yes	Yes
	2	<1km Away	No, but there were LGs/ LSs less than 1km away
	3	>1km Away	No, but there were LGs/ LSs between 1-5km away, No, but there were LGs/ LSs more than 5km away
	4	Outside of Patrol Hours	No it was outside patrolled hours
	5	No	No, Unsure
**What were you doing at the time of the rescue?**	1	Swimming	Swimming, Supervising kids swimming
	2	Non- powered watercraft	Surfing, Canoeing/kayaking/ paddle boarding
	3	Walking/running nearby	Walking/running nearby
	4	Sunbathing/ watching the water	Sunbathing/ watching the water
	5	Boating	Boating
	6	Dining nearby	Dining nearby
	7	Other	Other, Rock fishing of fishing from rocks, Beach/ land based fishing
**Were there people present when the rescue took place?**	1	Lots of people	Yes, it was busy with lots of people around
	2	Only a few people	Yes, it was busy with lots of people around
	3	Nobody	Nobody was around except for me and the person in trouble, I can’t remember

**What was your relationship to the person at the time of the rescue?** *['Other' was recoded into the appropriate category]*	1	Unknown	Stranger
	2	Known	Spouse/ Partner, Friend, Parent, Brother/ Sister, Child, Grandchild, Grandparent, Uncle/ Aunt, Cousin
**Did the person/s have any flotation devices with them at the time?**	1	No	No, I don't know
	2	Yes	Yes
**Did you use any devices to help you during the rescue, such as a surfboard or life jacket?**	1	No	No
	2	Yes	Yes- a surfboard, Yes- a life jacket
	3	Other	Other
**How was the person/s after the rescue?**	1	Did not need assistance	Ok- they could walk away, I can't remember
	2	Required medical assistance	Required First Aid, Required CPR, Required and Ambulance
**What would you do differently next time?**	1	Grab a flotation device	
	2	Tell someone else/ call for assistance/ organise more help	
	3	Grab a flotation device AND tell someone/get help	*[Open Answer Responses]*
	4	Calm the person down	
	5	Ensure parental supervision	
	6	Assist earlier	
	7	Escape the water/rip in a different way	
	8	Prevention- warn the people before they get in to trouble	*[Open Answer Responses]*
	9	Other	

APPENDIX 3. Actions that the bystanders indicated they would do differently next time they performed a rescue.

	Waterway Location			Gender		Water Safety Training at time of rescue		
	Coastal	Pool	Inland	Male	Female	None	Bronze Medallion	Water trained
Grab a flotation device	4	1	1	4	2	2	1	3
Tell someone else	9	1	0	3	7	3	1	6
Grab a flotation device AND tell someone	1	0	0	1	0	0	0	1
Calm the person down	2	0	0	1	1	1	1	0
Ensure parental supervision	3	2	0	3	2	3	1	1
Assist earlier	2	1	1	3	1	2	0	2
Escape the water/rip in a different way	3	0	1	4	0	0	1	3
Prevention- warn the people before they get in to trouble	2	0	0	2	0	0	1	1
Other	7	0	1	5	3	5	0	3
