# Supplementary material for: Characteristics of aquatic rescues undertaken by bystanders in Australia
Source: PLoS One. 2019 Feb 14;14(2):e0212349. doi: 10.1371/journal.pone.0212349 (PMC6375621; doi:10.1371/journal.pone.0212349)
Supplement: S3 Appendix — (PDF) [file pone.0212349.s003.pdf]

Appendix 3. Actions that the bystanders indicated they would do differently next time they performed a rescue.

	**Waterway Location**			**Gender**		**Water Safety Training at time of rescue**		
	Coastal	Pool	Inland	Male	Female	None	Bronze Medallion	Water trained
Grab a flotation device	4	1	1	4	2	2	1	3
Tell someone else	9	1	0	3	7	3	1	6
Grab a flotation device AND tell someone	1	0	0	1	0	0	0	1
Calm the person down	2	0	0	1	1	1	1	0
Ensure parental supervision	3	2	0	3	2	3	1	1
Assist earlier	2	1	1	3	1	2	0	2
Escape the water/rip in a different way	3	0	1	4	0	0	1	3
Prevention- warn the people before they get in to trouble	2	0	0	2	0	0	1	1
Other	7	0	1	5	3	5	0	3
